# A population-based estimation of breast cancer recurrence in northeast Italy with administrative healthcare databases

**DOI:** 10.1016/j.breast.2025.104487

**Published:** 2025-05-01

**Authors:** Fabiola Giudici, Federica Toffolutti, Stefano Guzzinati, Francesco Schettini, Marina Bortul, Silvia Francisci, Manuel Zorzi, Sara De Vidi, Daniela Pierannunzio, Luigino Dal Maso

**Affiliations:** a Cancer Epidemiology Unit, Centro di Riferimento Oncologico di Aviano (CRO) IRCCS, Aviano, Italy; b Epidemiological Department, Azienda Zero, Padua, Italy; c Translational Genomics and Targeted Therapies in Solid Tumors, August Pi i Sunyer Biomedical Research Institute (IDIBAPS), Barcelona, Spain; d Medical Oncology Department, Hospital Clinic of Barcelona, Barcelona, Spain; e Faculty of Medicine and Health Sciences, University of Barcelona, Barcelona, Spain; f Division of General Surgery, Department of Medical and Surgical Sciences, Breast Unit, Cattinara University Hospital, Trieste, Italy; g National Centre for Disease Prevention and Health Promotion, National Institute of Health, Rome, Italy

**Keywords:** Breast cancer, Stage at diagnosis, Recurrence, Competing risk, Survival

## Abstract

**Background/Aim:**

Information on the long-term frequency of recurrence is of paramount importance for the increasing number of women living several years after breast cancer (BC) diagnosis and for their caregivers.

The study aims to estimate the cumulative incidence of recurrence until 10 years after diagnosis in Italian women diagnosed with BC using population-based cancer registries.

**Methods:**

Women diagnosed with stage I to III BC during 2004–2010 from Friuli Venezia Giulia and Veneto (Italy) cancer registries were included (n = 5825). Recurrence status after a disease-free period was ascertained through individual-level linked databases using treatment or procedure codes from claims. Cumulative incidence of recurrence was calculated in the presence of competing risks (second cancer or death).

**Results:**

During a median follow-up of 13.5 years, 1522 out of 5825 women experienced a recurrence with an estimated 10-years cumulative incidence of 20.8 % (95 %CI:19.7–21.8 %), decreasing from 23.7 % in 2004–2006 to 18.5 % in 2007–2010. Women younger than 40 years (40.5 %), with stage III (41.8 %) and triple-negative BC (32.5 %) showed a higher 10-year incidence of recurrence.

At 10 years after a BC diagnosis, 83.9 % of women were alive: 67.5 % without any cancer-related events, 12.4 % after recurrence and 4.0 % after second primary cancer. 10-years survival was higher than 90 % for women with stage I BC and 58.1 % for those with stage III (3.2 % and 27.3 % deaths after recurrence, respectively).

**Discussion:**

This Italian study provide detailed population-based information on the incidence of recurrence and other outcomes after BC and may be replicated in other Italian and European areas.

## Highlights

• At 10 years after breast cancer diagnosis, 84 % of Italian women were alive, 68 % without any cancer related event.
• Cumulative recurrence incidence was 13 % at 5 years and 21 % at 10 years since breast cancer diagnosis.
• The use of population-based data is a feasible and cost-effective approach to study long-term outcomes in breast cancer.
• The methodology could be applied to other European countries with similar healthcare systems and to other cancers.

## Abbreviation

BC: Breast Cancer
CIs: Confidence Intervals
CR: Cancer Registry
ER: Estrogen Receptor
FVG: Friuli Venezia Giulia
HDD: Hospital Discharge Data
HER2: Human Epidermal growth factor Receptor 2
HR: Hormonal Receptor
ICD-10: International Classification of Disease 10th version
ICD-9 CM: International Classification of Disease, 9h revision, Clinical Modification
OPS: Outpatients Service database
PR: Progesterone Receptor
TN: Triple Negative

## 1 Introduction

The number of women living for many years after a breast cancer (BC) diagnosis is increasing in Europe [1,2] as elsewhere [3]. Even if most of them have a life expectancy comparable to that of the general population [4], recurrences related to the primary tumour are observed even many years after diagnosis.

Knowing the probability of second BC events (i.e., loco-regional recurrences, metastases and second primary BCs) is of paramount importance in improving the patient's quality of life [5], for decisions about their treatment and follow-up, and from the public health perspective to identify research priorities and health services needed in a definite population [6,7].

At present, knowledge about cancer recurrence (usually defined as the return of disease after a disease-free interval) and progression (i.e., any transition to a more advanced disease state without a disease-free interval) [8] is based primarily on the intensive follow-up of select patient populations at specialized settings, such as those enrolled in longitudinal research studies or clinical trials [9].

The interest in intermediate outcomes after a BC diagnosis is growing and population-based cancer registries could provide real-world information to clinicians and patients about treatment effectiveness and prognosis [[9], [10], [11], [12], [13]]. Unfortunately, population-based registry collection of outcomes other than vital status and cause of death is extremely resource-consuming and not collected by most registries [12,13]. As a consequence, several different approaches have been explored to fill this gap of knowledge, with the limitation of high heterogeneity among them, in terms of reproducibility, completeness of data sources used, and definition of recurrence. The methods to identify recurrence included statistical modelling [14], artificial intelligence algorithms [[15], [16], [17], [18]], and the use of population-based administrative healthcare databases [[19], [20], [21], [22], [23]].

Using the latter approach, the primary aim of this study is to estimate BC recurrences up to 10 years after diagnosis in Italian women through individual record linkage of two population-based cancer registries with their respective regional administrative health care databases.

## 2 Material and Methods

### 2.1 Study population

The study population was extracted from two regional population-based cancer registries located in the northeast of Italy (i.e., Friuli Venezia Giulia -FVG- and Veneto).

The International Classification of Diseases, 10th version (ICD-10) was used to identify malignant/invasive BC cases (C50) and the TNM (7th edition) to define the stage at diagnosis. We collected standard immunohistochemical tumour markers describing the surrogate intrinsic BC subtypes [24,25], namely hormonal receptors (HR) (i.e., estrogens [ER]/progesterone receptors [PR]) and human epidermal growth factor receptor 2 (HER2). These markers were collected for at least 80 % of cases allowing for the classification of BCs into three groups: HR-positive (ER or PR positive and HER2 negative), HER2 positive (HER2 positive and any ER/PR status) and Triple Negative (TN) (both HR and HER2 negative).

All women with BC diagnosed during 2004–2010 were included in FVG Cancer Registry, while Veneto Cancer Registry contributed only in a selected area which represented half of the Veneto population, with cases diagnosed in 2009, the only year when biomarker information was systematically collected (i.e., for at least 80 % of cases). Follow-up was conducted until December 31, 2021, to have a follow-up period at least of 10 years for all patients.

Among the identified patients with BC (n = 9407), women with the following characteristics were excluded from the cohort: history of other cancer diagnoses before BC (n = 572) or synchronous cancers (n = 141) (except for non-melanoma skin cancer, ICD10: C44), BC diagnosis by autopsy or death certificate only (n = 37), no survival time (n = 17) and death within 6 months after BC diagnosis (n = 13). We further excluded patients with missing stage (n = 537) or *de novo* metastatic BC (Stage IV, n = 252), and patients older than 74 years (n = 2013). The cohort enrolled to estimate recurrences included 5825 women (Supplementary Fig. 1).

To retrieve information regarding recurrences, BC data extracted from the cancer registries were linked to two administrative health care individual-record regional databases: the hospital discharge data (HDD) and the outpatient services database (OPS). The HDD provides information on the date of admission and discharge as well as diagnostic and procedure codes using the International Classification of Diseases, 9th Revision, Clinical Modification (ICD-9 CM) for each admission, while the OPS contains information on the date and type of service also coded according to ICD-9 CM. The choice to use as administrative health databases HDD and OPS, is based on the assumption that almost all women who will develop a recurrence will then receive chemotherapy and/or radiotherapy treatment or have hospitalization for treatment/procedures related to BC recurrence. As regards the pathology reports, many recurrences do not have a pathological confirmation, so this source, although it can certainly be useful to describe in detail the biological characteristics of the second event, is not essential for the identification of the recurrence.

Among the codes related to health services after a BC diagnosis [26], procedures and diagnoses that could indicate any type of recurrence (i.e., local, regional, distant recurrence or second primary BC) have been selected (Supplementary Table 1).

Recurrence is defined as the earliest of the following four indicators occurring during the follow-up period (Supplementary Fig. 2):

1. Any treatment or procedure code indicating restarting new chemotherapy;
2. Any treatment or procedure code indicating restarting new radiotherapy;
3. Breast surgery treatment code (mastectomy) or hospital admission code indicating a malignant breast neoplasm;
4. Any hospital admission code indicating a secondary malignant neoplasm.

The recurrence date was defined as the first date of any of the previous events.

The assessment of BC recurrence started from 12 months (for HER2 negative BC) or 24 months (for HER2 positive BC) since the date of diagnosis of BC. This surveillance period takes into account both the duration of primary treatment changing by the surrogate subtypes [27,28], and a disease-free period of 6 months after the end of primary treatment, according to the follow-up guidelines [29] which recommended follow-up visits every 3/6 months during the first 3 years post-treatment. The frequency of the follow-up changed over the years, but is unlikely to have an impact on the probability of recurrence [30]. This approach, with delayed entry at one/two years after BC diagnosis, was performed to reduce the treatment selection bias of survivors and to misclassification [31]. Sensitivity analysis was also performed using a cut- off of 12 months for all BC subtypes **(**Supplementary Table S4)

### 2.2 Statistical analysis

The frequency of recurrence up to 5 and 10 years (with 95 % Confidence Intervals, CIs) was estimated using the cumulative incidence function, overall and by period of diagnosis (2004–2006, 2007–2010), age (20–39, 40–49, 50–69, 70–74 years), surrogate subtype (HR+, HER2+, TN) and stage of disease (I, II, III). Cumulative incidence of recurrence was estimated in a competing risks framework which considered second tumours and deaths from all causes as competing events [32]. Women were followed up from the date of BC diagnosis until the following event (whichever came first): recurrence (event), second primary cancer (competing event), death for all causes (competing event), emigration (censoring event), or last follow-up, December 31, 2021 (censoring event). Given the descriptive nature of the study, predictive risk estimation models were not implemented. However, the time-dependent behaviour of prognostic factors related to recurrence event, has been evaluated. All analyses were carried out using the SAS software (version 9.4) and the free software R [33] particularly the “survival” [34] and “cmprsk” [35] packages.

## 3 Results

Clinical and pathological features of the 5825 women diagnosed with non-metastatic invasive BC in northeast Italy between 2004 and 2010 are illustrated in Table 1.

**Table 1:** Characteristics of women with a first diagnosis of non-metastatic breast cancer in 2004–2010. FVG-Veneto Italian Cancer Registries.

Characteristic	N = 5825 women	
	N	%
**Period of diagnosis**		
2004–2006	2540	43.6 %
2007–2010	3285	56.4 %
**Age**		
20–39	321	5.5 %
40–49	1152	19.8 %
50–59	1487	25.5 %
60–69	2098	36.0 %
70–74	767	13.2 %
**Tumour size**		
<20 mm	4031	69.2 %
≥20 mm	1686	28.9 %
Not available	108	1.9 %
**Lymph nodal status**		
N0	3488	59.9 %
N1	1516	26.0 %
N2	388	6.7 %
N3	247	4.2 %
Not available	186	3.2 %
**Histology**		
Ductal	4651	79.8 %
Lobular	685	11.8 %
Adenocarcinoma	152	2.6 %
Cystic, mucinous and serous	105	1.8 %
Other	232	4.0 %
**HR Status**^a^		
Positive	4850	83.3 %
Negative	800	13.7 %
Not available	175	3.0 %
**Stage**		
I	2971	51.0 %
II	2085	35.8 %
III	769	13.2 %
**Histology**		
Ductal	4651	79.8 %
Lobular	685	11.8 %
Adenocarcinoma	152	2.6 %
Cystic, mucinous and serous	105	1.8 %
Other	232	4.0 %
**Immunohistochemical Profile**		
HR+/HER2-	3852	66.1 %
HER2+	842	14.5 %
TN	455	7.8 %
Not available	676	11.6 %
**Vital Status at the end of Follow-up**^b^		
Alive	4410	75.7 %
Dead	1415	24.3 %
**Vital Status at 10 years of Follow-up**		
Alive	4886	83.9 %
Dead	939	16.1 %

Abbreviation: FVG, Friuli Venezia Giulia; ER: estrogen receptor; PR: progesterone receptor HR: hormone receptor; HER2, human epidermal growth factor receptor 2; TN: triple negative, i.e. HR- and HER2-.

a include tumours with mixed HR expression (either estrogen or progesterone receptor positive).

b Median follow-up of 13.5 years (interquartile range: 11.5–15.4).

Half of them (50.8 %) were aged less than 60 years, 69.2 % had primary tumours <2 cm, 79.8 % had ductal/no special type invasive histology, 59.9% had no nodal involvement and 51.0 % had early Stage I BC.

Full immunohistochemical data were available for 5149 women (88.4 %). Among them, 74.8 % were HR+/HER2-, 16.4 % HER2+, and 8.8 % TN. Women were followed for a total of 63562 person-years (py) and 83.9 % of them were alive at 10 years after diagnosis.

Over a median follow-up of 13.5 years (Interquartile Range: 11.5–15.4), 1522 women (26.1 %) developed a recurrence identified by codes related to chemotherapy (5.2 %), radiotherapy (4.1 %), breast surgery or hospitalization for malignant neoplasm of the breast (13.5 %), and secondary malignant neoplasm (3.3 %).

**Fig. 1:**
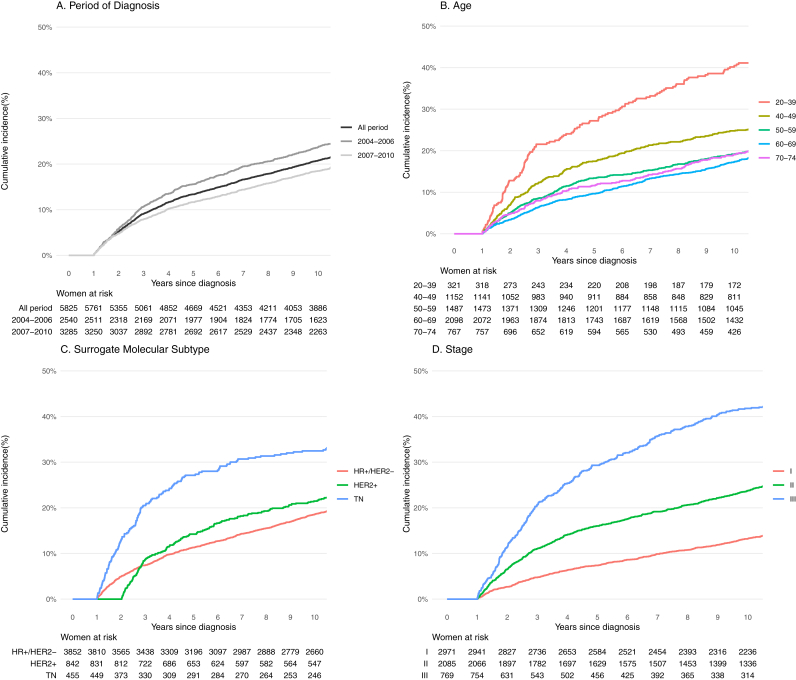
Cumulative Incidence function curves^a^ for women with non-metastatic breast cancer according to age, period of diagnosis, surrogate molecular subtype and stage of disease in 2004–2010. FVG-Veneto Italian Cancer Registries ^a^adjusted for competing risks.

The cumulative incidence of recurrence is shown in Fig. 1 for the most important prognostic factors, considering death for all causes and the incidence of a second primary tumor before evidence of recurrence as a competing risk (Supplementary Fig. S3).

The overall cumulative incidence of recurrence at 5 and 10-years were 13.4 % (95 %CI:12.5 %–14.3 %) and 20.8 % (95 %CI:19.7 %–21.8 %), respectively (unless otherwise stated, the 10-years estimate of cumulative incidence will be reported subsequently). The cumulative incidence decreased from 23.7 % in 2004–2006 to 18.5 % in 2007–2010 (Fig. 1A and Table 2). It was higher for women aged less than 40 years (40.5 %) than those aged 40–49 years (24.8 %) or older BC patients showing percentages below 20 % (Fig. 1B). BC recurrence also differed substantially across surrogate subtypes (Table 2) with a 10-years cumulative incidence of 32.5 % among TN (27 % after 5 years) and about 20 % among women with HR + or HER2+ tumours (11.3 % and 14.5 % after 5 years, respectively).

**Table 2:** Cumulative incidence of recurrence^a^ in women with a first diagnosis of non-metastatic breast cancer in 2004–2010. FVG-Veneto Italian Cancer Registries.

	**N°**	Cumulative Incidence (95 % Confidence intervals)	
		**At 5years**	At 10 years
**All Cohort**	**5825**	**13.4 % (12.5 %-14.3 %)**	**20.8 % (19.7 %-21.8 %)**
**Period of Diagnosis**			
2004–2006	2540	15.6 % (14.2 %–17.0 %)	23.7 % (22.1 %–25.4 %)
2007–2010	3285	11.7 % (10.6 %–12.8 %)	18.5 % (17.2 %–19.8 %)
**Age**			
20–39	321	27.2 % (22.4 %–32.2 %)	40.5 % (35.1 %–45.8 %)
40–49	1152	17.5 % (15.3 %–19.7 %)	24.8 % (22.3 %–27.3 %)
50–59	1487	13.4 % (11.7 %–15.2 %)	19.2 % (17.2 %–21.2 %)
60–69	2098	9.6 % (8.4 %–11.0 %)	17.3 % (15.7 %–19.0 %)
70–74	767	11.6 % (9.6 %–14.0 %)	19.1 % (16.4 %–21.9 %)
**Surrogate Subtype**			
HR+/HER2-	3852	11.3 % (10.3 %–12.3 %)	18.5 % (17.3 %–19.8 %)
HER2+	842	14.2 % (12.0 %–16.7 %)	21.5 % (18.8 %–24.3 %)
TN	455	27.1 % (23.1 %–31.3 %)	32.5 % (28.2 %–36.8 %)
**Stage**			
I	2971	7.4 % (6.5 %–8.4 %)	13.2 % (12.0 %–14.5 %)
II	2085	16.0 % (14.5 %–17.6 %)	23.8 % (21.9 %–25.6 %)
III	769	29.3 % (26.2 %–32.6 %)	41.8 % (38.3 %–45.2 %)

a adjusted for competing risks.

We also observed a change in the pattern of recurrence during the follow-up, consistent with the significantly time-dependent effect of the surrogate molecular subtype: whereas in TN BC the incidence of recurrence was higher in the first 5 years after diagnosis and would be quite stable thereafter (low probability of events), women with HR + subtype continued to have a risk of recurrence throughout all the observed period (Fig. 1C and Supplementary Table S5 which reported the conditional probabilities of recurrence). The stage at diagnosis sharply affected the probability of subsequent events, with 13.2 % of 10-years recurrences for patients diagnosed at stage I, 23.8 % at stage II, and 41.8 % at stage III (Fig. 1D) (at 5 years, 7.4 %, 16.0 % and 29.3 %, respectively). Crude recurrence rate until the end of follow-up for BC patients per 1000 person-years, overall and according to relevant prognostic factors were reported in Supplementary Table 2.

At 10 years after BC diagnosis, 83.9 % of women were alive, including 12.4 % after a recurrence and 67.5 % without any evidence of cancer-related events (Table 3).

**Table 3:** Follow-up status at 10 years since diagnosis, for women with a first diagnosis of non-metastatic breast cancer in 2004–2010. FVG-Veneto Italian Cancer Registries.

	Alive events free	Alive after recurrence	Alive after second tumour	Dead without recurrence^a^	Dead after recurrence	Dead after second tumour
**All Cohor**t	**3929(67.5 %)**	**722 (12.4 %)**	**235 (4.0 %)**	**316 (5.4 %)**	**487 (8.4 %)**	**136 (2.3 %)**
**Age**						
20–39	176 (54.8 %)	86 (26.8 %)	8 (2.5 %)	8 (2.5 %)	43 (13.4 %)	0 (0.0 %)
40–49	827 (71.8 %)	213 (18.5 %)	23 (2.0 %)	16 (1.4 %)	71 (6.2 %)	2 (0.2 %)
50–59	1056 (71.0 %)	164 (11.0 %)	70 (4.7 %)	52 (3.5 %)	121 (8.1 %)	24 (1.6 %)
60–69	1442 (68.7 %)	187 (8.9 %)	99 (4.7 %)	126 (6.0 %)	177 (8.4 %)	67 (3.2 %)
70–74	428 (55.8 %)	72 (9.4 %)	35 (4.7 %)	114 (14.9 %)	75 (9.8 %)	43 (5.6 %)
**Stage**						
I	2258 (76.0 %)	299 (10.1 %)	140 (4.7 %)	107 (3.6 %)	96 (3.2 %)	71 (2.4 %)
II	1352 (64.8 %)	313 (15.0 %)	77 (3.7 %)	114 (5.5 %)	181 (8.7 %)	48 (2.3 %)
III	319 (41.5 %)	110 (14.3 %)	18 (2.3 %)	95 (12.4 %)	210 (27.3 %)	17 (2.2 %)
**Surrogate Subtype**						
HR+/HER2-	2683 (69.7 %)	452 (11.7 %)	165 (4.3 %)	193 (5.0 %)	265 (6.9 %)	94 (2.4 %)
HER2+	561 (66.6 %)	96 (11.4 %)	33 (3.9 %)	46 (5.5 %)	83 (9.9 %)	23 (2.7 %)
TN	250 (54.9 %)	65 (14.3 %)	17 (3.7 %)	37 (8.1 %)	82 (18.0 %)	4 (0.9 %)

a includes progression without disease free period >6 months.

Overall, 371 women (6.3 %) developed a second primary cancer before any recurrence of BC and, among them, 4.0 % were alive. The sites of the second primary tumours are reported in Supplementary Table 3. Stratifying patients according to stage, at 10 years after diagnosis more than 90 % of women with stage I BC were alive (3.2 % deaths after recurrence), 58.1 % when diagnosed at stage III (27.3 % deaths after recurrence). Although 84.1 % of younger women (<40 years) were alive 10 years after a BC diagnosis, this age group showed the highest proportion of deaths after recurrence (13.4 %) compared with those aged 40–49 years (6 %) and older women (8.9 %). The frequency of deaths after recurrence for women with TN tumours (18.0 %) was twice as high as for other subtypes (6.9% for HR+/HER2- and 9.9% for HER2+ BC) (Table 3). Supplementary Table S6 also shows the women's probability of being alive with recurrence at 7 and 10 years, conditioned they were alive with a recurrence at 2 or 5 years after diagnosis.

Overall, 939 (16.1 %) women died during the 10-years follow-up, 57.9 % of them as the consequence of their BC.

## 4 Discussion

This study estimated the frequency of recurrence after BC diagnosis (i.e., all second BC events, including loco-regional recurrences, metastases and second primary BCs) in northestern Italy up to 10 years after diagnosis at a population level and according to several prognostic factors. Cumulative recurrence incidence was 13.4 % (95 %CI:12.5 %–14.3 %) at 5 years and 20.8 % (95 %CI:19.7 %–21.8 %) at 10 years since diagnosis. In Italy as elsewhere, the risk of developing a breast recurrence varied, depending on the characteristics of the patients (i.e., recurrence 2 times more frequent for women younger than 40 years, compared to later ages) and of the tumours (i.e., 3 times higher probability of recurrence in those with stage III BC vs stage I, or 1.5 times higher if the tumour was TN vs HR+/HER2-). Notably, 83.9 % of BC women were alive 10 years after diagnosis and more than two-thirds (67.5 %) were alive without any cancer-related event. In particular, for women who lived after a diagnosis of stage I BC, the proportion of deaths after BC recurrence (3.2 %) was lower than deaths unrelated to BC (2.4 % after another tumour and 3.6 % after other non cancer causes). These results can be seen in the light of proposals to redefine the high-risk period as one in which cancer mortality is the leading cause of death [36,37]. The decline in the recurrence between the two diagnostic periods (from 23.7 % to 18.5 %) is consistent with the introduction of adjuvant trastuzumab (in Italy in 2006), which has significantly reduced recurrences in HER2+ disease (which were most frequent in the first 2–5 years) [38]. Moreover, since the early 2000s, aromatase inhibitors have been the standard adjuvant endocrine therapy for postmenopausal women with HR + BC, replacing tamoxifen in many cases. Hormone receptor positive disease tends to recur especially beyond 5 years making the effect of the introduction more delayed and maybe more visible in 2006–2010 for people treated earlier [39]. In addition, a possible effect of the reduced incidence of recurrence could be due to the introduction of the mammography screening program in Friuli Venezia Giulia at the end of 2005, which led to a consequent downstaging.[40]

Notwithstanding several studies were published to investigate the BC recurrence, most of them have a heterogeneous study design, various follow-up durations, and different definition of recurrence. The importance of standardizing the collection of data on cancer recurrence at a population level is not only stressed by the European Network of Cancer Registries [8] but also recently the Lancet Breast Cancer Commission called for the worldwide collection of high-quality cancer registry data on cancer relapses [41].

Our approach follows the methodology proposed by prior studies [[18], [19], [20], [21], [22], [23]] using population based administrative health care databases linked at the individual level.

Among population-based cohorts with a median follow-up longer than 10 years, few studies considered both locoregional and distant metastasis: Geurts et al. [42] using data from Netherlands Cancer Registry showed that 20 % of BC patients diagnosed in 2003 had a recurrence, while a subsequent Dutch study including patients diagnosed in 2005 [13] reported a percentage of recurrence of 20.5 % until 10 years after diagnosis. A Germany registry-based [43] estimated a 10-year cumulative incidence of 16 % and investigated also the pattern of recurrence according to surrogate subtype, reporting a 10-year cumulative incidence for HR+/HER2- (14 %), HER2+ (21.7 %) and TN (25.6 %). Our estimates are consistent with these results, even if frequency of recurrence for all patients and for HR+/HER2- subgroup are a little lower in Germany (15.9 % and 14.0 %) than in Italy (20.8 % and 18.5 %). This difference could be explained by the fact that the German cohort exclusively consisted of women who had received local surgery with free resection margin and were therefore at reduced risk of developing locoregional relapses [44]. Ess et al. [45] (Switzerland) likewise evaluated the impact of BC subtypes on recurrence and, such as in our study, the authors demonstrated that the risk of recurrence varied considerably according to subtype: for the HR + cases the risk continued to increase during the entire follow-up period, while for TN cancers it increased rapidly in the first years and remained quite stable after 5 years since diagnosis. Ess et al. [45], differently from our study, reported that patients with HR-/HER2+ subtypes had worse prognosis than TN breast malignancy including women diagnosed in the period 2003–2005 when HER2-targeted therapy with trastuzumab was not yet approved for early-stage disease.

Late recurrences remain a relevant issue for patients with HR + BC: the importance of at least 10 years of follow-up when studying recurrences was confirmed in a recent large pooled analysis involving 151 trials [9]. For women with ER + disease, the risk of distant recurrence persisted at a constant rate beyond 5 years, whereas most recurrences for HR- cancers occurred in the first 5 years. Similarly, Pan et al. [46], considering 88 trials (50 % of whom had a period of diagnosis similar to our study) showed that late recurrences remained a relevant issue for patients with luminal breast cancer.

The subtype-specific recurrence times (early for TN vs. late for HR+) call for risk-stratified follow-up care for BC patients [47]. Our findings suggest that women with TN disease relapsed mainly in the first 2–5 years and can be considered “cured” after this period, having an almost negligible risk of recurrence. For them, an earlier reduction of follow-up could be proposed (e.g., after 5 years). On the other hand, for HR+ BCs follow-up should continue up to 10 years and beyond, a strategy that is currently being implemented [29].

Few population-based cancer registries investigated the incidence of recurrence in the young women with BC. In our Italian cohort, a higher frequency of recurrence (40.5 %) was estimated below age 40 years in comparison to older patients (i.e., <20 % after 50 years). These results are consistent with those reported in Spanish and Swiss studies [48,49]. This very high recurrence rates in young women make it necessary to improve treatment strategies, which has happened or is happening in the last decade (i.e., immunotherapy with neoadjuvant chemotherapy in TN stage II-III, double anti HER2 blockade with trastuzumab and pertuzumab in HER2+ stage II-III, adjuvant CDK4/6 inhibitors in high-risk HR+/HER2- [29]).

It is important to bear in mind that the diagnostic period of our study (2004–2010) may not reflect the treatment patterns of BC patients in subsequent decades. Consequently, caution is necessary when considering the generalizability of our findings, in particularly for TN BCs whose real improvement is seeing especially in the last few years with the introduction of carboplatin in neoadjuvant [50] and with introduction of pembrolizumab [51].

### 4.1 Limitation and strengths

We acknowledge several important limitations. The clinical chart manual review of BC cases cannot be performed and, using administrative healthcare databases, we are not able to disentangle local and regional from distant recurrences. Moreover, misclassification of second breast primary tumor as recurrence is also possible [52], supporting the need of more detailed data collection by cancer registries. In addition, it is possible that a small proportion of women developed an untreated recurrence without any hospitalization or outpatients’ treatment, resulting in an underestimation of the recurrence rate. However, this bias was unlikely, given the exclusion of patients aged 75 years or older (i.e., with frequent comorbidities [53]), who will require a specific study tailored to investigate the incidence of recurrence. The occurrence of false positives is an issue in cancer algorithms as codes related to the primary tumor may be misinterpreted as a recurrence. Nevertheless, tailoring the cut-off times of the surveillance period according to the surrogate subtype and the delayed-entry approach [54] should ensure that the therapies for the primary tumor have been completed. We were unable to assess the prognostic significance of clinical biomarkers or lifestyle factors which may influence the incidence of recurrence [55] because this information could not be determined from cancer registries. In order to definitively assess the accuracy of the proposed method, however, an external validation of the algorithm through comparison with the results of the manual review of medical records is necessary where possible [12]. This validation could allow for stratification of analyses according to different types of second events, identifying specific recurrence codes. Finally, an update using more recent years of diagnosis or a longer follow-up (15 years) are needed in Italy or countries with similar health care systems.

Our study has also several strengths. The completeness and accuracy of the cancer registries' data on incidence and survival in Italy represent one of those [1,4,56]. Moreover, the duration of follow-up (median 13.5 years) and the application of competing risk models contributed to provide accurate estimates of long-term survival and recurrence.

The length and the quality of vital status follow-up ascertainment up to 10 years after BC diagnosis, both for women who developed a recurrence and for those who did not, is another strength of the study.

The comprehensiveness and high quality of administrative healthcare databases are crucial for recurrence estimation. Therefore, the availability of almost two decades of complete and detailed hospitalization and outpatient data in northeast Italy has proven to be able to study events following a cancer diagnosis [26,57]. Reassuringly, the recurrence patterns observed are in line with results from studies which analyzed recurrence by different BC subtypes [58], strongly suggesting that the methodology is reliable.

Finally, the data management is compliant with the General Data Protection Regulation (GDPR): we adopted a privacy-preserving record linkage, using a pseudonymized personal identifier number. Consequently, the presented methodology could be applied to estimate recurrence from population-based cancer registry data at the national and European level and also for other cancers, with comparable results [59,60] to support proper health care policies on cancer prevention and management.

## 5 Conclusions

This population-based population cohort shows that up to 10 years after diagnosis about 80 % of Italian women with non-metastatic BC remained recurrence-free and less than 10 % die from their malignancy.

The results of this study will help monitor costs to the health care system and provide detailed and highly relevant information, based on the risk of recurrence and other long-term outcomes after BC, useful for patients themselves, health care professionals, and policy makers.

**Table:** Glossary

Population-based cancer registry	it collects data on every person with cancer in a defined population, usually comprising people resident in a well-defined geographical region.
Cancer Recurrence	the return of cancer after a disease-free period
Second breast cancer events	recurrences and second primary breast cancers
Competing risk	event whose occurrence precludes the occurrence of the primary event of interest.
Cumulative Incidence	risk that an individual will experience an event during a specified period of time: it is calculated as the number of new events or cases of disease divided by the total number of individuals in the population at risk for a specific time interval.

## CRediT authorship contribution statement

**Fabiola Giudici:** Writing – review & editing, Writing – original draft, Methodology, Formal analysis, Conceptualization. **Federica Toffolutti:** Software, Methodology, Data curation, Conceptualization. **Stefano Guzzinati:** Writing – review & editing, Software, Data curation. **Francesco Schettini:** Writing – review & editing, Methodology. **Marina Bortul:** Writing – review & editing, Methodology. **Silvia Francisci:** Writing – review & editing, Project administration. **Manuel Zorzi:** Writing – review & editing, Methodology. **Sara De Vidi:** Writing – review & editing. **Daniela Pierannunzio:** Writing – review & editing, Methodology. **Luigino Dal Maso:** Writing – review & editing, Writing – original draft, Project administration, Methodology, Funding acquisition, Conceptualization.

## Ethics

The Italian legislation identifies Cancer Registries (CR) as collectors of personal data for surveillance purposes without explicit individual consent. The approval of a research ethics committee is not required, since this descriptive study was conducted without any direct or indirect intervention on patients.

## Funding

This work was supported by the 10.13039/501100005010Italian Association for Cancer Research (AIRC) (Grant no. 28893); 10.13039/501100003196Italian Ministry of Health (Ricerca Corrente) (no grant number). This project is co-funded by the 10.13039/501100000780European Union, JAPreventNCD (Grant no. 101128023). Views and opinions expressed are however those of the author(s) only and do not necessarily reflect those of the European Union or European Health and Digital Executive Agency (HaDEA). Neither the European Union nor HaDEA can be held responsible for them.

The funding sources had no involvement in the study design, in the collection, analysis, and interpretation of data, in the writing of the report, and in the decision to submit the article for publication.

## Declaration of competing interests

F. Schettini reports honoraria from Novartis, Gilead, Veracyte and Daiichy-Sankyo for educational events/materials, advisory fees from Pfizer and Veracyte, and travel expenses from Novartis, Gilead and Daiichy-Sankyo. The other authors have nothing to declare.
