# Supplementary material for: A population-based estimation of breast cancer recurrence in northeast Italy with administrative healthcare databases
Source: Breast. 2025 May 1;82:104487. doi: 10.1016/j.breast.2025.104487 (PMC12133715; doi:10.1016/j.breast.2025.104487)

**Supplementary appendix**

Supplement to: Giudici F. Toffolutti F., Guzzinati S., et al. A population-based estimation of breast cancer recurrence in northeast Italy with administrative healthcare databases

**Corresponding author:**

Fabiola Giudici, Cancer Epidemiology Unit, Centro di Riferimento Oncologico di Aviano (CRO) IRCCS, Via Franco Gallini 2, 33081 Aviano (PN), Italy, Tel: (+39) 0434 659354; Fax: (+39) 0434 659231, E-mail: [fabiola.giudici@cro.it](mailto:fabiola.giudici@cro.it) ORCID: 0000-0002-4160-3479

**Table of contents**

**Supplementary Table 1.** Hospital and Outpatient Administrative Codes (ICD9-CM) used to Identify Breast Cancer Recurrence………………………………………………………………………...................................3

**Supplementary Table 2**. Frequency of recurrences and Crude Recurrence Rate (per 1000 PY) until the end of follow-up for women with non-metastatic breast cancer by Period of Diagnosis, Age, Lymph node status, Surrogate Subtype and Stage in 2004-2011. FVG and Veneto Italian Cancer Registries..................................6

**Supplementary Table 3.** Number and percentage of incident second primary tumours before recurrence at 10 years since BC diagnosis in 2004-2010. FVG-Veneto Italian Cancer Registries…...……………...……….7

**Supplementary Table 4.** Sensitivity analysis: cumulative incidence of recurrence in women with a first diagnosis of non-metastatic breast cancer in 2004-2010. FVG-Veneto Italian Cancer Registries…..................7

**Supplementary Table 5.** Probabilities to be alive at 7 and 10 years conditioned to be alive and recurrence-free at 2 and 5 years respectively, by Stage of Disease and Surrogate Molecular profile in women with a first diagnosis of non-metastatic breast cancer in 2004-2010. FVG-Veneto Italian Cancer Registries…………….8

**Supplementary Table 6.** Probabilities to be alive with recurrence at 7 and 10 years conditioned to be alive and at 2 and 5 years respectively, by Stage of Disease and Surrogate Molecular profile in women with a first diagnosis of non-metastatic breast cancer in 2004-2010. FVG-Veneto Italian Cancer Registries.……….……8

**Supplementary Figure 1.** Flow-chart of Cohort Selection……………………………………………………9

**Supplementary Figure 2**. Schematic overview of recurrence identification procedure……….………....… 10

**Supplementary Figure 3**. Cumulative Incidence function curves for women with non-metastatic breast cancer according to competing events, in 2004-2010. FVG-Veneto Italian Cancer Registries...………....… 11

**Supplementary Table 1**

**Hospital and Outpatient Administrative Codes (ICD9-CM) used to Identify Breast Cancer Recurrence**

| **Intervention** | **ICD9-CM CODE** | **DESCRIPTION** | **Database** |
| --- | --- | --- | --- |
| **Chemotherapy** | **PROCEDURE** |  | -Hospital Discharge Database (SDO-Schede di Dimissione Ospedaliera)  -Outpatient Services Database (Prestazioni Ambulatoriali) |
|  | 99.25 | Injection or infusion of cancer chemotherapeutic  substance |  |
|  | 99.28 | Injection or infusion of biological response modifier ÝBRM¨ as an antineoplastic agent |  |
|  | 00.10 | Implantation of Chemotherapy agent |  |
|  | **DIAGNOSIS** |  | Hospital Discharge Database (SDO-Schede di Dimissione Ospedaliera) |
|  | V58.1 | Encounter for chemotherapy and immunotherapy for  neoplastic conditions |  |
|  | V58.11 | Encounter for antineoplastic chemotherapy |  |
|  | V58.12 | Antineoplastic immunotherapy |  |
|  | **ICD9-CM CODE** | **DESCRIPTION** |  |
| **Radiotherapy** | **PROCEDURE** |  | -Hospital Discharge Database (SDO-Schede di Dimissione Ospedaliera)  -Outpatient Services Database (Prestazioni Ambulatoriali) |
|  | 92.23 | Radioisotopic teleradiotherapy |  |
|  | 99.24 | Teleradiotherapy using photons |  |
|  | 92.25 | Teleradiotherapy using electrons |  |
|  | 92.26 | Teleradiotherapy of other particulate radiation |  |
|  | 92.27 | Implantation or insertion of radioactive elements |  |
|  | 92.28 | Injection or instillation of radioisotopes |  |
|  | 92.29 | Other radiotherapeutic procedure |  |
|  | **DIAGNOSIS** |  |  |
|  | V580 | Radiotherapy | Hospital Discharge Database (SDO-Schede di Dimissione Ospedaliera) |
|  | **ICD9-CM CODE** | **DESCRIPTION** |  |
|  | **PROCEDURE** |  |  |
|  | 85.23 | Subtotal mastectomy |  |
|  | 85.24 | Excision of ectopic breast tissue |  |

| **Surgery** | 85.25 | Excision of nipple | Hospital Discharge Database (SDO-Schede di Dimissione Ospedaliera) |
| --- | --- | --- | --- |
|  | 85.33 | Unilateral subcutaneous mammectomy with synchronous  implant |  |
|  | 85.34 | Other unilateral subcutaneous mammectomy |  |
|  | 85.35 | Bilateral subcutaneous mammectomy with synchronous  implant |  |
|  | 85.36 | Other bilateral subcutaneous mammectomy |  |
|  | 85.41 | Unilateral simple mastectomy |  |
|  | 85.42 | Bilateral simple mastectomy |  |
|  | 85.43 | Unilateral extended simple mastectomy |  |
|  | 85.44 | Bilateral extended simple mastectomy |  |
|  | 85.45 | Unilateral radical mastectomy |  |
|  | 85.46 | Bilateral radical mastectomy |  |
|  | 85.47 | Unilateral extended radical mastectomy |  |
|  | 85.48 | Bilateral extended radical mastectomy |  |
|  | **ICD9-CM CODE** | **DESCRIPTION** |  |
| **Malignant neoplasm of female Breast** | 174.0 | Nipple and areola | Hospital Discharge Database (SDO-Schede di Dimissione Ospedaliera) |
|  | 174.1 | Central portion |  |
|  | 174.2 | Upper-inner quadrant |  |
|  | 174.3 | Lower-inner quadrant |  |
|  | 174.4 | Upper-outer quadrant |  |
|  | 174.5 | Lower-outer quadrant |  |
|  | 174.6 | Axillary tail |  |
|  | 174.8 | Other specified sites of female breast |  |
|  | 174.9 | Breast (female), unspecified |  |
| **Secondary and unspecified malignant neoplasm of lymph nodes** | 196.0 | Lymph nodes of head, face, and neck | Hospital Discharge Database (SDO-Schede di Dimissione Ospedaliera) |
|  | 196.1 | Intrathoracic lymph nodes |  |
|  | 196.2 | Intra-abdominal lymph nodes |  |
|  | 196.3 | Lymph nodes of axilla and upper limb |  |
|  | 196.5 | Lymph nodes of inguinal region and lower limb |  |
|  | 196.6 | Intrapelvic lymph nodes |  |
|  | 196.8 | Lymph nodes of multiple sites |  |
|  | 196.9 | Site unspecified Lymph nodes NOS |  |
|  |  |  |  |
| **Secondary malignant** | 197.0 | Secondary malignant neoplasm of the lung |  |
|  | 197.1 | Secondary malignant neoplasm of the mediastinum |  |

| **neoplasm of respiratory and digestive systems** | 197.2 | Secondary malignant neoplasm of the pleura | Hospital Discharge Database (SDO-Schede di Dimissione Ospedaliera) |
| --- | --- | --- | --- |
|  | 197.3 | Secondary malignant neoplasm of other respiratory  organs |  |
|  | 197.4 | Secondary malignant neoplasm of the small intestine,  including duodenum |  |
|  | 197.5 | Secondary malignant neoplasm of the large intestine and  rectum |  |
|  | 197.6 | Secondary malignant neoplasm of the retroperitoneum  and peritoneum |  |
|  | 197.7 | Secondary malignant neoplasm of the liver |  |
|  | 197.8 | Secondary malignant neoplasm of the other digestive  organs and spleen |  |
| **Secondary malignant neoplasm of other specified sites** | 198.0 | Secondary malignant neoplasm of the kidney | Hospital Discharge Database (SDO-Schede di Dimissione Ospedaliera) |
|  | 198.1 | Secondary malignant neoplasm of other urinary organs |  |
|  | 198.2 | Secondary malignant neoplasm of the skin |  |
|  | 198.3 | Secondary malignant neoplasm of the brain and spinal  cord |  |
|  | 198.4 | Secondary malignant neoplasm of the other parts of the  nervous system |  |
|  | 198.5 | Secondary malignant neoplasm of the bone and bone  marrow |  |
|  | 198.6 | Secondary malignant neoplasm of the ovary |  |
|  | 198.7 | Secondary malignant neoplasm of the adrenal gland |  |
|  | 198.8 | Secondary malignant neoplasm of other sites |  |
|  | 198.82 | Secondary malignant neoplasm of the genital organs |  |
|  | 199.0 | Malignant neoplasm disseminated |  |
|  | 199.1 | Malignant neoplasm NOS |  |

ICD9-CM = International Classification of Diseases 9th Revision codes: Centers for Disease Control and Prevention. International Classification of Diseases, 9th Revision, Clinical Modification (ICD9-CM). https://[www.cdc.gov/nchs/icd/icd9cm.htm.](http://www.cdc.gov/nchs/icd/icd9cm.htm) Published 2016. Accessed 4 Nov 2020

**Supplementary Table 2: Frequency of recurrences and Crude Recurrence Rate (per 1000 PY) until the end of follow-up^1^ for women with non-metastatic breast cancer by Period of Diagnosis, Age, Lymph node status, Surrogate Subtype and Stage in 2004-2011. FVG and Veneto Italian Cancer Registries**

| Variable | Number of  Any Recurrence  (N, %) | Total Person Years (PY) | Crude Recurrence Rate  per 1000 PY (95% CI) |
| --- | --- | --- | --- |
|  |  |  |  |
| All Cohort | **1522 (26.1%)** | **63562** | **23.9 (22.8-25.2)** |
|  |  |  |  |
| Period of Diagnosis |  |  |  |
| 2004-2006 | 774 (30.5%) | 29575 | 26.2 (24.4-28.0) |
| 2007-2010 | 748 (22.8%) | 33987 | 22.0 (20.5-23.6) |
|  |  |  |  |
| Age |  |  |  |
| 20-39 | 150 (46.7%) | 3046 | 49.2 (41.8-57.6) |
| 40-49 | 341 (29.6%) | 12792 | 26.7 (23.9-29.6) |
| 50-59 | 382 (25.7%) | 16840 | 22.7 (20.5-25.0) |
| 60-69 | 483 (23.0%) | 23399 | 20.6 (18.9-22.5) |
| 70-74 | 166 (21.6%) | 7485 | 22.2 (19.0-25.7) |
|  |  |  |  |
| Surrogate Subtype |  |  |  |
| HR+ / HER2- | 936 (24.1%) | 42915 | 21.8 (20.4-23.2) |
| HER2+ | 220 (28.8%) | 9183 | 24.0 (20.9-27.3) |
| TN | 159 (35.6%) | 4214 | 37.7 (32.2-43.9) |
|  |  |  |  |
| Stage |  |  |  |
| I | 555 (18.7%) | 34977 | 15.9 (14.6-17.2) |
| II | 612 (29.4%) | 22307 | 27.4 (25.3-29.7) |
| III | 355 (46.2%) | 6278 | 56.5 (50.9-62.7) |

^1^Cases diagnosed in 2004-2010, follow-up 31/12/2021, median length of FU 13.5 years.

**Supplementary Table 3: Number and percentage of incident second primary tumours before recurrence at 10 years since BC diagnosis in 2004-2010. FVG-Veneto Italian Cancer Registries**

| Second Primary Tumour Before Recurrence | N | % |
| --- | --- | --- |
| C18-C21-Colon | 68 | 18.33% |
| C33-C34-Lung | 50 | 13.48% |
| C54-Uteri Corpus | 40 | 10.78% |
| C43-Skin Melanoma | 25 | 6.74% |
| C82-85, C96-NH Lymphoma | 22 | 5.93% |
| C25-Pancreas | 21 | 5.66% |
| C64-C66, C68-Kidney | 17 | 4.58% |
| C67- C67, D9.0, D30,3, D41.4 Bladder | 17 | 4.58% |
| C73-Thyroid | 16 | 4.31% |
| C16-Stomach | 16 | 4.31% |
| C56-Ovary | 12 | 3.23% |
| C47,C49-Soft Tissue | 11 | 2.96% |
| Other (Freq <10) | 56 | 15.09% |
| Total | 371 | 100.0% |

**Supplementary Table 4. Sensitivity analysis: cumulative incidence of recurrence^a^ in women with a first diagnosis of non-metastatic breast cancer in 2004-2010. FVG-Veneto Italian Cancer Registries**

| Period of Diagnosis |  | Cumulative incidence (95% CI) at 5 years | |  | Cumulative incidence (95% CI) at 10 years | |
| --- | --- | --- | --- | --- | --- | --- |
|  | n |  |  |  |  |  |
| 2004-2006 | 2540 | 17.2% | (15.7%-18.7%) |  | 24.9% | (23.3%-26.7%) |
| 2007-2010 | 3285 | 13.8% | (12.7%-15.0%) |  | 20.2% | (18.9%-21.6%) |
|  |  |  |  |  |  |  |
| Age |  |  |  |  |  |  |
| 20-39 | 321 | 31.2% | (26.2%-36.3%) |  | 44.0% | (38.5%-49.3%) |
| 40-49 | 1152 | 20.5% | (18.2%-22.9%) |  | 27.5% | (25.0%-30.2%) |
| 50-59 | 1487 | 15.0% | (13.2%-16.8%) |  | 20.5% | (18.5%-22.6%) |
| 60-69 | 2098 | 11.0% | (9.7%-12.4%) |  | 18.2% | 16.5%-19.8%) |
| 70-74 | 767 | 13.0% | (10.8%-15.5%) |  | 20.0% | (17.2%-22.9%) |
|  |  |  |  |  |  |  |
| Surrogate Molecular profile |  |  |  |  |  |  |
| HR+ | 3850 | 11.2% | (10.3%-12-3%) |  | 18.3% | (17.1%-19.5%) |
| HER2+ | 842 | 28.1% | (25.1%-31.2%) |  | 34.6% | (31.4%-37.8%) |
| TN | 455 | 26.5% | (22.5%-30.6%) |  | 31.1% | (26.9%-35.4%) |
|  |  |  |  |  |  |  |
| Stage |  |  |  |  |  |  |
| I | 2971 | 8.5% | (7.6%-9.6%) |  | 14.3% | (13.0%-15.6%) |
| II | 2085 | 18.5% | (16.8%-20.2%) |  | 25.6% | (23.7%-27.5%) |
| III | 769 | 32.7% | (29.4%-36.0%) |  | 44.2% | (40.7%-47-7%) |

^a^ Adjusted for competing risks with a cut-off of 12 months as starting time for the surveillance period

**Supplementary Table 5.** **Probabilities to be alive at 7 and 10 years conditioned to be alive and recurrence-free at 2 and 5 years respectively, by Stage of Disease and Surrogate Molecular profile** **in women with a first diagnosis of non-metastatic breast cancer in 2004-2010. FVG-Veneto Italian Cancer Registries**

|  | Conditional Probabilities to be alive with recurrences | |
| --- | --- | --- |
|  | **Year 7\|2** | **Year 10\|5** |
| Stage |  |  |
| I | 95.9% | 94.6% |
| II | 92.0% | 91.9% |
| III | 78.9% | 80.6% |
|  |  |  |
| Surrogate Molecular Profile |  |  |
| HR+/HER2- | 93.2% | 92.1% |
| HER2+ | 87.2 % | 93.3% |
| TN | 82.8% | 93.3% |

**Supplementary Table 6.**  **Probabilities to be alive with recurrence at 7 and 10 years conditioned to be alive and at 2 and 5 years respectively, by Stage of Disease and Surrogate Molecular profile in women with a first diagnosis of non-metastatic breast cancer in 2004-2010. FVG-Veneto Italian Cancer Registries**

|  | Conditional Probabilities to be alive with recurrences | |
| --- | --- | --- |
|  | **Year 7\|2** | **Year 10\|5** |
| Stage |  |  |
| I | 76.1% | 58.8% |
| II | 61.2% | 51.2% |
| III | 30.8% | 23.3% |
|  |  |  |
| Surrogate Molecular Profile |  |  |
| HR+/HER2- | 63.2% | 49.1% |
| HER2+ | 20.8% | 38.5% |
| TN | 29.2% | 34.8% |

**Supplementary Figure 1: Flow-chart of Cohort Selection**

**Invasive Breast Cancer (ICD-10: C50) RT-FVG_Veneto**

**2004-2010**

N=9407

Analysed cohort after exclusions

**N=5,825**

Exclusions

- Previous history of invasive cancers (excluded ICD10: C44), n=572
- Synchronous cancer (<90 days from incidence BC), n=141

n= 8694

Exclusions

- DCO or autoptic diagnosis, n= 37
- No follow-up, n=17
- Deceased within six months post-diagnosis, n=13

n= 8627

Exclusions

- Stage IV, n=252
- Stage Missing, n=537

n= 7838

Exclusions

- Age >74, n=2013

**Supplementary Figure 2: Schematic overview of recurrence identification procedure**


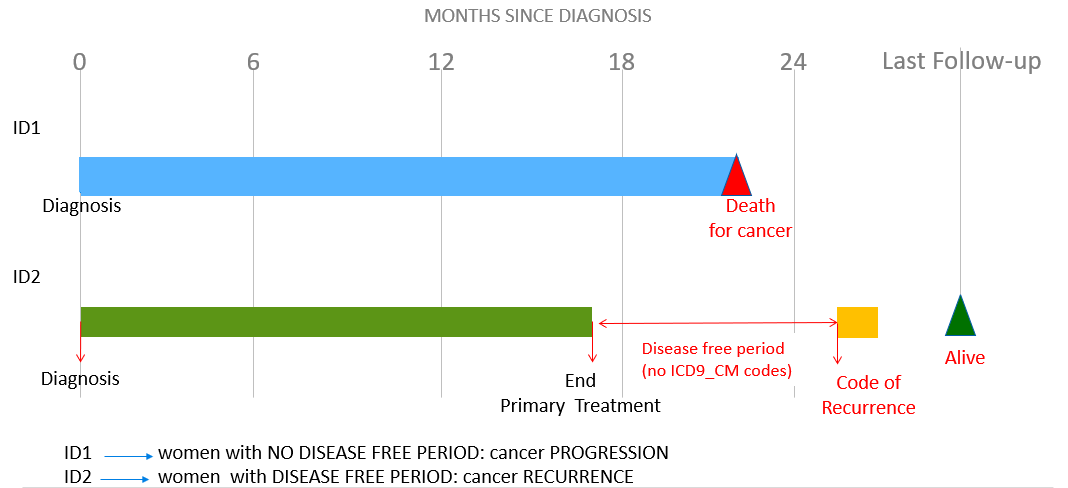


Note. Delayed-entry approach: assessment of BC recurrence started following a delayed entry at 12 months post-cancer diagnosis to reduce survivor treatment selection bias. Only for HER2+ breast cancer patients, due to the duration of targeted therapy, the assessment of cancer recurrence began at 24 months since the BC diagnosis.

**Supplementary Figure 3:** **Cumulative Incidence function curves for women with non-metastatic breast cancer according to competing events, in 2004-2010. FVG-Veneto Italian Cancer Registries**


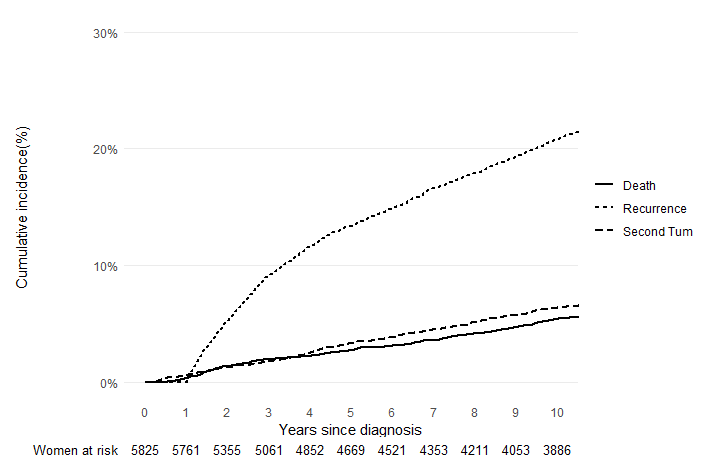

Supplement: Multimedia component 1 [file mmc1.docx]
